# Community Interaction Co-limitation: Nutrient Limitation in a Marine Microbial Community Context

**DOI:** 10.3389/fmicb.2022.846890

**Published:** 2022-05-25

**Authors:** Catherine Bannon, Insa Rapp, Erin M. Bertrand

**Affiliations:** ^1^Department of Biology and Institute for Comparative Genomics, Dalhousie University, Halifax, NS, Canada; ^2^Marine Biogeochemistry Division, GEOMAR Helmholtz Centre for Ocean Research Kiel, Kiel, Germany

**Keywords:** nutrient co-limitation, microbial community interactions, cobalamin, nitrogen fixation, marine microbes, primary producers

## Abstract

The simultaneous limitation of productivity by two or more nutrients, commonly referred to as nutrient co-limitation, affects microbial communities throughout the marine environment and is of profound importance because of its impacts on various biogeochemical cycles. Multiple types of co-limitation have been described, enabling distinctions based on the hypothesized mechanisms of co-limitation at a biochemical level. These definitions usually pertain to individuals and do not explicitly, or even implicitly, consider complex ecological dynamics found within a microbial community. However, limiting and co-limiting nutrients can be produced *in situ* by a subset of microbial community members, suggesting that interactions within communities can underpin co-limitation. To address this, we propose a new category of nutrient co-limitation, community interaction co-limitation (CIC). During CIC, one part of the community is limited by one nutrient, which results in the insufficient production or transformation of a biologically produced nutrient that is required by another part of the community, often primary producers. Using cobalamin (vitamin B_12_) and nitrogen fixation as our models, we outline three different ways CIC can arise based on current literature and discuss CIC’s role in biogeochemical cycles. Accounting for the inherent and complex roles microbial community interactions play in generating this type of co-limitation requires an expanded toolset – beyond the traditional approaches used to identify and study other types of co-limitation. We propose incorporating processes and theories well-known in microbial ecology and evolution to provide meaningful insight into the controls of community-based feedback loops and mechanisms that give rise to CIC in the environment. Finally, we highlight the data gaps that limit our understanding of CIC mechanisms and suggest methods to overcome these and further identify causes and consequences of CIC. By providing this framework for understanding and identifying CIC, we enable systematic examination of the impacts this co-limitation can have on current and future marine biogeochemical processes.

## Introduction

Major nutrients (N and P) and micronutrients (Fe, Co, Zn, Mn, B_1_, B_12_) have been found to directly limit phytoplankton growth alone or in combination (Bertrand et al., 2007; Moore et al., 2013; Paerl et al., 2017; Wu et al., 2019; Browning et al., 2021), thereby impacting marine food webs and ocean productivity. Nutrient stoichiometries and molecular measurements in the global ocean suggest that primary production in large parts of the surface ocean may be subject to co-limitation and stress (defined as a physiological response to nutrient scarcity, Moore et al., 2013) due to simultaneous deficiencies in multiple nutrients (Moore et al., 2013; Saito et al., 2015; Browning et al., 2017). Co-limitation has been suggested to be a common condition in heterogenous microbial communities because of different nutrient demands between microbial groups and taxa-specific abilities to adapt to low nutrient conditions (Danger et al., 2008). Cellular stoichiometry for micronutrients, such as trace metals, can vary by more than two orders of magnitude, with variability between taxa as well as between growth conditions and nutrient gradients in the ocean (Moore et al., 2013; Twining and Baines, 2013; Twining et al., 2021). Cellular stoichiometries for macronutrients show less variation but deviate significantly from the Redfield ratio for individual species (Ho et al., 2003; Garcia et al., 2018). Additionally, certain members of a phytoplankton community may have different abilities to access or preferences for nutrient pools, such as ligand-bound Fe (Hutchins et al., 1999) and forms of nitrogen [e.g., N_2_, NO_x_, NH_4_ or urea (dissolved organic nitrogen: DON)] (Moore et al., 2002; Collier et al., 2009, 2012). Comparatively little is known about the requirements for B-vitamins, though it is clear that eukaryotic phytoplankton and the majority of cyanobacteria require different forms of cobalamin (Helliwell et al., 2016; Heal et al., 2017), and that different species can satisfy their thiamine requirements using different thiamine-related compounds (Paerl et al., 2017, 2018a,2018b).

Co-limitation is commonly divided into 3 types: (I) independent co-limitation, (II) biochemical substitution co-limitation and (III) biochemically dependent co-limitation (Saito et al., 2008). Independent co-limitation occurs when two entirely independent nutrients are simultaneously drawn down to limiting conditions either for a single organism or, in a community context, different parts of the community are limited by different nutrients. This type can either occur as true co-limitation or as serial limitation, where the secondary limiting nutrient only becomes limiting when the limitation by the primary limiting nutrient has been relieved. Biochemical substitution co-limitation occurs when one limiting nutrient can be substituted for the other limiting nutrient or the addition of one nutrient can partly relieve the nutrient stress imposed by the lack of another limiting nutrient and vice versa (Saito et al., 2008). Biochemically dependent co-limitation occurs when one nutrient is required for acquisition of another nutrient at low concentrations (Saito et al., 2008; Moore et al., 2013). These definitions describe scenarios where two nutrients are simultaneously limiting phytoplankton growth but can be further extended to three or more nutrients. These different categories of nutrient limitation have been subject to many studies and reviews to date (e.g., see Saito et al., 2008; Harpole et al., 2011; Moore et al., 2013).

Currently, the mechanisms describing these types of co-limitation do not consider complex interactions that arise when a limiting nutrient is produced *in situ* by a subset of the microbial community. Such conditions have been identified in the ocean (Bertrand et al., 2015) and have the potential to respond to change much differently than other types of co-limitation. We suggest this necessitates an additional definition that acknowledges the interdependencies and exchange of essential materials in microbial communities. To this end, we introduce the concept of community interaction co-limitation (CIC). CIC describes a community where multiple limiting nutrient cycles are affected by one another through the interactions between groups within the community. In other words, one part of the community is limited by one nutrient, which results in the insufficient production or transformation of a biologically produced nutrient (BPN) that is required by another part of the community, often primary producers. This results in a community that is limited by multiple nutrients because of the interactions among different microbial groups present. Unlike other categories of nutrient co-limitation, community interaction co-limitation cannot be observed in a monoculture and is not relevant in the context of an individual. Some examples of CIC are given in Table 1 and include limitation of bacterial vitamin production by a specific nutrient, which results in the limitation of the phytoplankton community that requires this vitamin but lacks the ability to synthesize it. Another example is the limitation of diazotrophs by the availability of iron and/or phosphorus in a nitrogen depleted region, resulting in nitrogen limitation of the non-diazotrophic community.

**TABLE 1:** Examples of community interaction co-limitation.

Examples for CIC	Biologically produced nutrient	Producers	Nutrient limiting production of biologically produced nutrient	Consumers of biologically produced nutrient	Select references
Limited availability of one or more nutrients limits B_12_ production by bacteria and archaea, resulting in phytoplankton growth being limited by B_12_	B_12_	Select bacteria and archaea	Iron, nitrogen, labile carbon, cobalt	Eukaryotic phytoplankton	Bertrand et al., 2007, 2015; Gobler et al., 2007; Koch et al., 2011; Barber-Lluch et al., 2019
Limitation of nitrogen fixers (e.g., by P or Fe) resulting in limitation of non-diazotrophic phytoplankton by N	Fixed nitrogen (e.g., NO_x_, NH_4_)	Diazotrophs	Iron, phosphate, labile carbon	Non-diazotrophic primary producers	Mills et al., 2004
Limitation of B_1_ production resulting in co-limitation of B_1_ auxotrophs by B_1_ and N/C	B_1_	Select bacteria, archaea, and eukaryotic phytoplankton	Nitrogen, carbon	B_1_-auxotrophic phytoplankton (e.g., chlorophyta) and select bacteria	Gobler et al., 2007; Koch et al., 2012; Paerl et al., 2018b

Here we aim to describe the importance of considering community dynamics in the interpretation of nutrient limitation in the ocean. Some of the examples provided above (Table 1) have previously been classified as one of the three traditional types of co-limitation, e.g., iron and B_12_ co-limitation as type I, and iron limitation of nitrogen fixation as type III (Saito et al., 2008). These traditional classifications are of most utility when focusing on one species or dominant plankton group at a time. We are suggesting that these classifications are not invalid but, instead, are incomplete. Marine microbial communities are webs of interactions which control the communities’ structure, function, and resource availability – factors that influence the community’s productivity, composition, and contribution to global-scale processes.

Community interactions are commonly overlooked during experiments investigating resource limitation, perhaps because of the complexity required to sample and interpret them. Yet, community interactions are commonly overlooked during experiments investigating resource limitation, perhaps because of the complexity required to sample and interpret them. Yet, community interactions control nutrient availability and metabolic status of individual community members through the exchange of nutrients and metabolites via direct, associated relationships (in the phycosphere) or through cross feeding of public goods in bulk medium (Seymour et al., 2017; Pacheco et al., 2019). A central focus of microbial ecology is to identify mechanisms through which communities assemble in order to predict potential for change and how that change could influence community function (e.g., Zomorrodi and Segrè, 2017; Fu et al., 2020). Incorporating ideas about microbial evolution, such as the Black Queen Hypothesis (discussed in Section “Incorporating Ecological and Evolutionary Theory Into Community Interaction Co-limitation”), aids in identifying the principles that govern the evolution of microbial dependencies and “public good” nutrient production, which could underpin such instances of this co-limitation (Mas et al., 2016). Incorporating ecological and evolution theories into resource limitation studies will enable stronger interpretations of community-based feedback loops that control resource availability, which could provide a framework to predict the response of microbial communities (e.g., Mas et al., 2016; Zomorrodi and Segrè, 2017; Fu et al., 2020). Considering CIC as a fourth and separate type of co-limitation will enable researchers to systematically leverage findings from microbial ecology and evolutionary ecology, highlighting the impact microbial interactions have on larger biogeochemical cycles and generating new insights on patterns and trends of nutrient limitation in the ocean.

## Community Interaction Co-Limitation

### Cobalamin

While community interaction co-limitation can extend to other BPNs (Table 1), we use the micronutrient cobalamin (vitamin B_12_) to provide evidence for CIC because of its importance in microbial community interactions and potential to co-limit primary production in open ocean and coastal waters (Croft et al., 2005; Bertrand et al., 2007; Ramanan et al., 2016; Sokolovskaya et al., 2020). Cobalamin is a cobalt-containing organometallic micronutrient that’s structure was first elucidated in 1956 by Nobel laureate Dr. Dorothy Crowfoot Hodgkin (Hodgkin et al., 1956). It is a powerful cofactor that performs rearrangements and methylation during biochemical reactions (Banerjee and Ragsdale, 2003; Dowling et al., 2012). Cobalamin can only be synthesized *de novo* by select bacteria and archaea, and it is required by auxotrophic bacteria and an estimated 50% of eukaryotic phytoplankton (Martens et al., 2002; Croft et al., 2005). Auxotrophy in eukaryotic phytoplankton is believed to arise from cobalamin’s role as a cofactor for the enzyme methionine synthase (MetH), which is the final step in methionine synthesis (Croft et al., 2005; Helliwell et al., 2011). All eukaryotic phytoplankton appear to have a cobalamin dependent MetH, while a subset have an additional cobalamin independent one (MetE) that is less efficient and more costly to use (Bertrand et al., 2013). Cyanobacteria produce and use their own cobalamin-like molecule, pseudo-cobalamin, that is largely unavailable to eukaryotic algae but can be re-modeled into cobalamin by a subset of heterotrophic bacteria and a small group of algae, if the alpha ligand (DMB) is available (Helliwell et al., 2016; Ma et al., 2020). This discovery improved, and complicated, our understanding of the marine cobalamin cycle, which now must consider pseudo-cobalamin and remodelers as a potential source of cobalamin. Cobalamin has a significant history at the intersection between biological oceanography and microbial ecology, which has been reviewed (Sañudo-Wilhelmy et al., 2014; Helliwell, 2017) but we will briefly outline its role in algal–bacterial interactions and as a limiting micronutrient here because of its relevance to community interaction co-limitation.

Cobalamin has an important role in microbial interactions and is regularly considered as a model metabolite exchanged in beneficial microbial interactions (Ramanan et al., 2016). Auxotrophic phytoplankton can acquire cobalamin through sustaining obligate or facultative interactions with B_12_-producing bacteria and/or archaea or by uptake of the vitamin from its surrounding environment, referred to as scavenging (Amin et al., 2012; Kazamia et al., 2012; Bertrand et al., 2015). However, the strategy an organism uses to obtain sufficient cobalamin is likely affected by the availability of the nutrient or associated producer. The factors that influence methods of cobalamin acquisition and the regulation of cobalamin transfer or exchange between organisms are still poorly understood although relevant for understanding CIC. Interactions between cobalamin producers and consumers can be selected for and strengthened, in part, by the reciprocal transfer of nutrients and/or the active selection of beneficial bacteria through secondary metabolites (Kazamia et al., 2012; Grant et al., 2014; Shibl et al., 2020). In particular, the exchange of organic, labile carbon for cobalamin is relatively well documented, and it is expected that other nutrients, such as nitrogen species, amino acids, sulfonates, sugar derivatives or specific growth factors like indol-3-acetic acid could be exchanged as well (Amin et al., 2012; Ramanan et al., 2016; Durham et al., 2019).

Cobalamin is found at picomolar to sub-picomolar concentrations in the open ocean (Sañudo-Wilhelmy et al., 2014) and has already been shown to limit or co-limit phytoplankton communities in multiple regions of the ocean (e.g., coastal McMurdo Sound and the Ross Sea, Gulf of Alaska, coastal NE Atlantic) (Bertrand et al., 2007, 2015; Koch et al., 2011; Barber-Lluch et al., 2019). These studies investigated cobalamin limitation using bottle incubation nutrient addition experiments, occasionally layering omics approaches to investigate mechanisms of limitation (Bertrand et al., 2015). A few studies have directly measured the concentration of cobalamin(s) in the water column (Gobler et al., 2007; Heal et al., 2014). Determining potential for cobalamin limitation through examining stoichiometry is not yet feasible since cobalamin production rates, requirements, and quotas are still poorly defined. Cobalamin availability is thought to depend on the rate of photodegradation, remodeling, and balance of supply and demand (driven by B_12_ producers and B_12_ consumers). But, cobalamin production can be limited by other nutrients required for bacterial growth (iron or nitrogen) or cobalamin production (e.g., cobalt).

### Nitrogen Fixation

It is also useful to consider the more extensively studied example of nitrogen fixation in the context of CIC. Since a number of reviews summarizing the current advances in understanding marine nitrogen fixation are available (e.g., Sohm et al., 2011; Zehr and Capone, 2020), we will only briefly highlight some key findings on community interactions as it pertains to CIC. Nitrogen fixation is performed by diazotrophs and comprises an important source of fixed nitrogen for non-diazotrophs in the ocean, especially during periods of low fixed nitrogen availability (Karl et al., 2002). However, diazotrophic activity, and therefore nitrogen supply, can be limited by iron and phosphorous availability, generating a community that is co-limited by iron and/or phosphorous and nitrogen (Held et al., 2020). In many cases, diazotrophs, such as *Trichodesmium* spp., independently fix atmospheric carbon but it remains unclear if the diazotroph benefits in any way from the release/exchange of fixed nitrogen. Some diazotrophic cyanobacteria observed to be associated with haptophytes appear to lack the ability to fix atmospheric carbon, thereby relying on the supply of organic carbon from the haptophytes (Thompson et al., 2012). Carbon and nitrogen exchange between the two symbionts therefore relies on adequate growth conditions for both organisms. Several recent studies also suggest a direct influence of DOM availability on nitrogen fixation rates when (non-cyanobacterial) heterotrophs dominate the diazotrophic community (Bonnet et al., 2013; Rahav et al., 2015, 2016). The nutrients produced within these symbiosis could be available to the surrounding community through active or passive transfer and, as such, suggest that nitrogen fixation is well suited to be investigated through the lens of CIC.

## Cases of Community Interaction Co-Limitation

**FIGURE 1:**
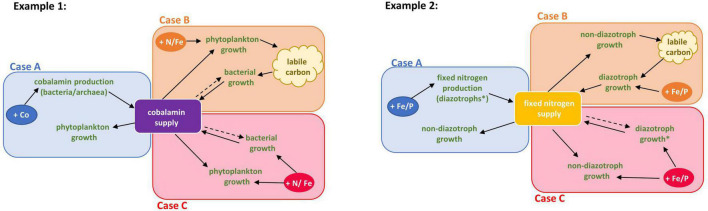
Cases of community interaction co-limitation modeled with cobalamin and nitrogen fixation. Solid arrows indicate that an increase in the resource or process where the arrow originates facilitates the process or production of the resource that the arrow is pointed toward. Dashed arrows indicate a potential influence of an increase in the resource (origin of arrow) on the process which resulted in its production (arrowhead) and thereby indicates potential resource consumption and/or competition. **Case A:** Biological production of a nutrient is restricted by the availability of nutrients required for the production of that nutrients. **Example 1:** Cobalt availability restricts cobalamin synthesis by heterotrophic bacteria which in turn limits phytoplankton by cobalamin. **Example 2:** Iron or phosphorus availability restricts nitrogen fixation by diazotrophs which limits fixed nitrogen supply to non-diazotroph growth. **Case B:** Biological production of a nutrient is limited by a factor which is produced by the consumer of the biological nutrient. **Example 1:** Nitrogen and/or iron co-limit phytoplankton growth which restricts dissolved organic carbon availability, limiting heterotrophic bacterial growth. This low bacterial growth then limits cobalamin production leaving the community co-limited by nitrogen or iron and cobalamin. **Example 2:** Iron or phosphorus availability restricts nitrogen fixation by diazotrophs which limits fixed nitrogen supply to non-diazotroph growth. This could result in a (co-)limitation of diazotrophs by DOC. **Case C:** Producers and consumers are limited by the same resource which further inhibits the biological production of a nutrient required by the consumers. **Example 1:** Both phytoplankton and heterotrophic bacteria are limited by iron (or another resource), which restricts cobalamin synthesis and could results in co-limitation of phytoplankton by cobalamin and iron. **Example 2:** Both diazotrophs or non-diazotrophs are limited by iron or phosphorus that constrains fixed nitrogen production which co-limits non-diazotrophs growth. *The diazotroph part of the community here could also represent or include close association between a diazotroph and an autotroph exchanging nutrients.

The production of a biological nutrient *in situ* can be restricted in a variety of ways, each of which have the potential to limit the growth of other community members (like phytoplankton) that require the BPN (Figure 1). Here we outline different cases of CIC based on the mechanisms that limit the availability of the BPN. The underpinning mechanism of CIC may determine how efficiently a microbial community can respond to changes in nutrient supply or recover from nutrient stress once the availability of the limiting nutrient increases. Categorizing CIC into specific cases clarifies mechanisms of limitation, potential metabolic interactions, and potential molecular responses for each CIC case. This will improve our ability to systematically predict the impact of CIC and allow development of targeted strategies to monitor spatiotemporal patterns in CIC.

Case A describes a scenario where the biological production of a nutrient (the BPN) is restricted by the availability of another nutrient required for BPN production. This results in the limitation of one part of the community by the BPN because the production is limited by another nutrient. An increase in the nutrient limiting the biological production likely increases the supply of the BPN immediately, or with a short time-lag. In Case B, the biological production of the nutrient is limited by a factor that is produced by the consumer of the BPN, which is restricted because of the limited availability of the other co-limiting nutrient. An increased supply of the additional co-limiting nutrient might not directly relieve the limitation by the BPN as the production remains limited by the factor produced by the consumer. Finally, in Case C, producers and consumers are limited by the same resource, which inhibits the production of a separate BPN that limits the consumer growth (Figure 1). This case would behave like Case A when the supply of the limiting nutrient is increasing, but consumers and producers may compete for the same resource.

We use examples, supported by literature, to describe the three cases of CIC in Figure 1. In Case A of cobalamin CIC, cobalamin biosynthesis by the producers is restricted by cobalt availability. Case B describes a community that is already limited by one or more nutrients, such as iron and/or nitrogen, which results in insufficient production of dissolved organic carbon. This restricts bacterial growth and cobalamin synthesis, which results in restricted cobalamin supply to phytoplankton. Case C represents a scenario where both phytoplankton and cobalamin producers are limited by the same nutrient (e.g., iron), thereby restricting cobalamin synthesis and consequently leaving the phytoplankton community co-limited by cobalamin and iron.

Nitrogen fixation CIC can also be classified into the proposed cases (Figure 1). Case A describes a community in which the diazotroph is limited by iron or phosphorous, resulting in nitrogen limitation of the non-diazotrophic community, rendering the overall community co-limited by nitrogen and iron or phosphorous. In Case B, nitrogen fixation by diazotrophs is limited by biologically produced compounds, resulting in nitrogen limitation of the phytoplankton community. The cause of such a scenario could be preceding iron or phosphorous limitation of diazotrophs, restricting nitrogen supply to the community and thereby DOM availability for the nitrogen fixers. After an increase in iron or phosphorous supply, the diazotrophs remain limited by DOM, impeding a fast recovery from the nutrient limited state. Case C describes a community in which both diazotrophs and non-diazotrophs are limited by the same nutrient, i.e., iron, resulting in co-limitation of the non-diazotrophs by nitrogen and iron. The close association between haptophytes and diazotrophic cyanobacteria may be described by Case B when only considering the two symbionts. However, looking at this from the community perspective which the CIC definition aims to achieve, one might consider the close association of haptophyte/diazotroph as one part of the community which supplies fixed nitrogen to other members of the community through grazing, remineralization, or passive release. If we assume that the haptophyte/diazotroph association is independent of DOM supply from other members of the community, the effect for the entire community would then classify as Case A or Case C depending on the mechanism of co-limiting nutrient (Figure 1).

We expect that other cases of CIC might become evident with additional research into auxotrophy and essential micronutrients, and that communities may experience a continuum, or a mix of more than one case occurring in the same community at any given time. Furthermore, there may also be links between CIC cases involving more than one BPN at the same time, for example, some cyanobacterial diazotrophs (UCYN-A) closely associated with haptophytes, are able to produce pseudocobalamin and might therefore be able to supply fixed nitrogen and cobalamin (after conversion) to their host (Koch et al., 2011; Muñoz-Marín et al., 2018; Barber-Lluch et al., 2019). Lesser explored community interactions (and therefore not included in Table 1) which could lead to CIC, might include exoenzymes which liberate inorganic nutrients for organisms other than those producing the enzymes (e.g., alkaline phosphatase; Luo et al., 2009). Phytoplankton might benefit from accessing these inorganic nutrients, rather than producing the enzymes required for the conversion themselves. Additionally, production of siderophores by select community members could lead to an increase in ‘new’ bioavailable iron by mobilizing particulate iron (Manck et al., 2022). The nutrient limiting the growth of siderophore producers could then possibly limit bioavailable iron delivery to the whole community, contributing to CIC. The details matter here though, as this siderophore-bound iron is not available to all community members equally (Hutchins et al., 1999; Sanchez et al., 2018; Sutak et al., 2020). Identifying microbial groups that rely on interactions to obtain BPNs will be important for investigations of additional mechanisms of CIC.

## Evidence of Community Interaction Co-Limitation in the Literature

**FIGURE 2:**
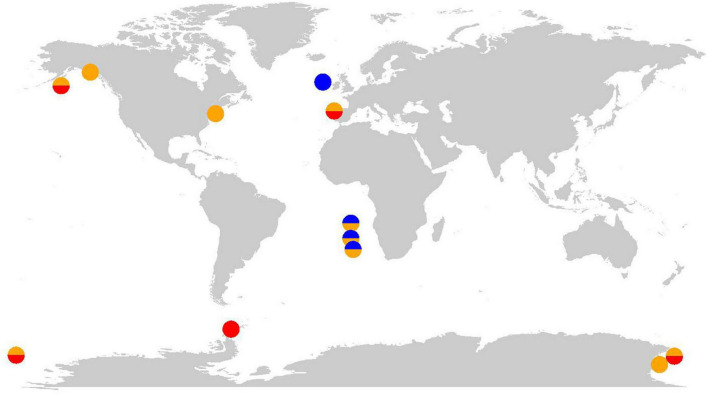
Global map indicating study sites where cobalamin co-limitation was observed. The color of circles indicates the presumed case or cases of CIC. Blue: Case A, orange: Case B, and red: Case C.

In the following, we describe nine studies that provide evidence that the *in situ* microbial community was experiencing at least one case of cobalamin CIC (Figure 2). Using the available data from these studies, we suggest the most likely case or cases of CIC (A-C) and highlight open questions which must be addressed to fully confirm the case type (Table 2). Generally, we suggest that the identification of CIC and underpinning mechanism requires demonstrating that the production of the BPN is limited by another resource and co-limiting phytoplankton growth. Traditionally, evidence for co-limitation has been gathered using ship-board bottle incubation bioassay experiments and evaluated based on phytoplankton growth responses to nutrient additions assessed by measuring chlorophyll *a*, photosynthetic efficiency (F_v_/F_m_), particulate organic carbon (POC) production, nutrient drawdown, and cell counts, often also assessing changes in community composition to some extent. However, bottle incubation bioassays alone are insufficient to identify the specific case of CIC (A-C) and describe any potential feedback loops of nutrient transfer in the community. Such identifications remain rare in the literature but may be accomplished using omics or other approaches that can resolve the response of different community members to nutrient manipulations (refer to section “Data gaps, uncertainties, and future directions” for expanded discussion).

**TABLE 2:** Instances of cobalamin community interaction co-limitation from literature.

Study site	Case of CIC	Evidence of co-limitation	Notes	Open questions	References
Sea ice edge of McMurdo sound	B	Addition of B_12_ and Fe led to increase in Chl *a* production and downregulation of iron and cobalamin deprivation indicators in phytoplankton and bacteria. Oceanospirillaceae ASP10-02A expressed organic compound acquisition genes and B_12_ production genes (see text).	No attempt to measure heterotrophic bacterial growth after nutrient additions.	Confirm limitation of B_12_ production by organic carbon availability?	Bertrand et al., 2015
Southern Ocean (Antarctic Peninsula)	C	Addition of B_12_, B_1_ and Fe together significantly enhanced phytoplankton growth. Addition of iron alone significantly increase bacterial production.	Addition of B-vitamins alone stimulated phytoplankton growth.	Were the bacteria stimulated by iron predominantly B_12_ consumers or producers?	Panzeca et al., 2006
Gulf of Alaska (coastal region)	B	NO_3_ + B_12_ addition resulted in the highest increase in > 2 μM phytoplankton and heterotrophic bacterial growth.	Addition of cobalamin alone did not increase phytoplankton growth but caused a shift in community composition. Addition of N alone did not increase heterotrophic bacterial growth. Most B_12_ uptake was likely by heterotrophic bacteria.	Were the stimulated bacteria B_12_ consumers or producers?	Koch et al., 2011
Gulf of Alaska (HNLC region)	B or C	Addition of B_12_ and Fe together significantly enhanced phytoplankton and heterotrophic bacteria growth.	Addition of B_12_ or Fe alone stimulated phytoplankton and bacterial growth. Most B_12_ uptake was likely by heterotrophic bacteria.	Were the stimulated bacteria B_12_ consumers or producers? Were the bacteria co-limited by iron and B_12_ or by production of organic matter?	Koch et al., 2011
Long Island embayment	B or C	Addition of B_12_ and NO_3_ together significantly enhanced phytoplankton growth.	No attempt to measure heterotrophic bacterial growth after nutrient additions. Addition of B_12_ alone stimulated > 5 μM phytoplankton growth.	B_12_ production limited by organic carbon or nitrogen availability?	Sañudo-Wilhelmy et al., 2006; Gobler et al., 2007
Ross Sea	B or C	Fe + B_12_ amendment experiment resulted in an increase in the phytoplankton growth.	Addition of B_12_ alone did not significantly stimulate phytoplankton growth. Most B_12_ uptake was by large phytoplankton. Iron addition led to a higher cobalt consumption, but cobalt did not limit phytoplankton growth or cobalamin production	Response of heterotrophic bacteria to nutrient additions?	Bertrand et al., 2007
North Atlantic Ocean (Spanish Coast) (October)	C	B_12_ + inorganic nutrients amendment experiments increased phytoplankton growth more than inorganic nutrient addition alone.	Addition of B_12_ or Fe alone stimulated phytoplankton growth. Increase in bacterial biomass during inorganic nutrient addition. Decrease in bacterial biomass during B_12_ addition.	Were the stimulated bacteria B_12_ consumers or producers? What warrants a decrease in bacterial biomass during B_12_ addition?	Barber-Lluch et al., 2019
North Atlantic Ocean (Spanish Coast) (March)	B*	B_12_ + inorganic nutrient amendment experiments increased phytoplankton growth more than inorganic nutrient addition alone.	No evidence of enhanced heterotrophic bacterial growth after inorganic nutrient additions.	What is limiting B12 production in bacteria?	Barber-Lluch et al., 2019
Eastern boundary of the South Atlantic gyre	A or B	Addition of nitrogen, iron and B_12_ or cobalt together significantly increased phytoplankton growth. This might suggest that B_12_ production was limited by cobalt.	No examination of heterotrophic bacterial growth after nutrient additions.	Is Co or B_12_ colimiting (or serially limiting) phytoplankton growth? Was cobalamin production limited by Co or DOC?	Browning et al., 2017
North Atlantic Ocean	A	Cobalt addition increased B_12_ production and phosphate and nitrate uptake. The concentration of dissolved B_12_ was positively correlated with Co concentrations, bacterial productivity, and phytoplankton biomass.	There was not an increase in phytoplankton biomass after Co addition	Change in community composition after nutrient additions?	Panzeca et al., 2008

Studies listed in Table 2 revealed increases in phytoplankton growth after the addition of cobalamin alone or in combination with other nutrients. Although the magnitude of responses to cobalamin is often lower than for other limiting nutrients, some studies have found B_12_ additions alone cause a very similar or even larger response in chlorophyll *a* concentration compared to the other co-limiting nutrients (Koch et al., 2011; Barber-Lluch et al., 2019), or observed a doubling in chlorophyll *a* after combined addition of B_12_ with nitrogen or iron compared to adding nitrogen or iron alone (Bertrand et al., 2007; Gobler et al., 2007). Additionally, these studies demonstrated a strong shift in community composition with the addition of cobalamin and revealed seasonal variability in cobalamin co-limitation. Most of the studies do not provide enough data to definitively determine the specific case of CIC. However, a good first indicator of what might be limiting cobalamin production is the response of heterotrophic bacterial growth to nutrient addition. This indicator, however, needs to be treated with caution as only a subset of bacteria are cobalamin producers and a larger fraction appears to be cobalamin consumers (Soto et al., in review^1^; Shelton et al., 2019).

Some studies included in Table 2 have additional limitations in terms of their ability to conclusively identify cases of CIC. For example, most studies did not quantify heterotrophic bacterial growth or did not assess the response to organic carbon addition, making it difficult to distinguish between Case B and C (Sañudo-Wilhelmy et al., 2006; Bertrand et al., 2007; Gobler et al., 2007; Koch et al., 2011). In the HNLC region in the Gulf of Alaska, an increase of phytoplankton and bacterial growth upon the addition of iron and B_12_ was recorded (Koch et al., 2011). However, with the data provided it is unclear if the observed bacterial growth was stimulated by the iron (indicating Case C) or by increased organic matter produced by actively growing phytoplankton (suggesting Case B). To determine the exact case of CIC present, researchers would have to demonstrate bacterial response to organic carbon addition or isolate the bacteria community independent of phytoplankton and then re-assess growth during iron addition. This is difficult, or impossible, to accomplish in complex communities. Other experimental approaches for determining the mechanism of CIC include the analysis of ‘omics data. For example, heterotrophic bacterial growth was not measured in a McMurdo Sound study (Bertrand et al., 2015), but elevated expression of organic carbon acquisition genes in a cobalamin producer, in response to iron addition, suggested that cobalamin production was likely limited by organic carbon availability once iron limitation of primary producers was alleviated.

The study that performed nutrient incubations off the Spanish Coast (Barber-Lluch et al., 2019), revealed seasonal variability in cobalamin co-limitation. Specifically, this study demonstrated a shift from Case B in March to Case C in October. From the data provided we concluded that the bacterial community was not limited by inorganic nutrients in March (eliminating Case C), in contrast to October when bacterial biomass increased upon addition of inorganic nutrients (potentially indicating Case C). Since cobalt availability is usually elevated in coastal regions due to inputs from continental shelf sediments (Noble et al., 2012; Tagliabue et al., 2018), it is unlikely that cobalt is a limiting nutrient. Therefore, we suggest that cobalamin production was most likely limited by a factor produced by the phytoplankton community in March, e.g., DOC, representing Case B.

Evidence for Case A was present in two studies (Panzeca et al., 2008; Browning et al., 2017). At the Eastern Boundary of the South Atlantic gyre, additions of nitrogen and iron with either cobalt or B_12_ increased phytoplankton growth over the combined addition of nitrogen and iron alone (Browning et al., 2017). Three different CIC interpretations are consistent with the available data. The first would suggest that cobalt is serially limiting phytoplankton growth and B_12_ addition only enhances growth through the indirect addition of cobalt contained in the B_12_ molecule, which must be re-packaged and used in ionic form in other enzymes. The second possibility would be that B_12_ production is limited by cobalt, and cobalt addition thereby enhances phytoplankton growth indirectly through the stimulation of B_12_ production, representing Case A. However, it is also possible that cobalt and B_12_ were both serially limiting phytoplankton growth and that cobalamin producers were limited by DOC, representing Case B. Clear identification of B_12_ stress of phytoplankton growth could have been achieved through the assessment of biomarkers that indicate cobalamin stress of phytoplankton (e.g., MetE), and the limiting factor for cobalamin production (cobalt or DOC) could have been assessed using multi-omic approaches. Panzeca et al. (2008) observed a correlation between cobalt and vitamin B_12_ concentrations, phytoplankton biomass and bacterial production in the North Atlantic Ocean. Although a clear confirmation of CIC is not available due to the lack of incubation experiments assessing phytoplankton biomass responses, it provides evidence for the presence of cobalt limitation of cobalamin production (Case A).

Despite numerous studies on marine nitrogen fixation, sufficient information from experiments that can identify CIC cases involving nitrogen fixation in the ocean are scarce. This is largely due to slow growth rates of many nitrogen fixers (Landolfi et al., 2015) and thereby slow response to nutrient additions, which makes a direct assessment of nutrient limitation of diazotrophs in bottle incubations challenging. Hence, only a few studies are available showing enhanced diazotroph growth after the addition of phosphorus, iron, or DOC while simultaneously demonstrating that phytoplankton growth was limited by nitrogen (Mills et al., 2004; Moisander et al., 2012). A study in the eastern tropical North Atlantic demonstrated phytoplankton growth to be primarily limited by nitrogen, whereas iron and phosphorous additions in the absence of added nitrogen did not influence phytoplankton growth but significantly enhanced nitrogen fixation rates (Mills et al., 2004), hence suggesting the presence of Case A. However, the effect of DOC addition was not tested in this study and DOC may have been additionally limiting. The observed increase in nitrogen fixation after addition of iron and phosphorous in the absence of enhanced phytoplankton growth does not suggest a major limitation by DOC. Another study in the South Pacific demonstrated an increase in several nitrogen fixers after the addition of iron alone and in combination with organic carbon, at stations where phytoplankton growth was limited by nitrogen (Moisander et al., 2012), suggesting the occurrence of Case A and Case B of CIC. However, iron additions contained EDTA and hence they could not rule out that EDTA may have acted as an additional carbon source and enhanced nitrogen fixation instead of iron. Additionally, some studies suggest the presence of CIC involving nitrogen fixation by demonstrating the nutrient limitation of diazotroph growth in nitrogen deplete conditions using lab-based experiments, cell quotas, transcriptomic and proteomic data (using biomarkers of nutrient stress), and observed correlations between nitrogen fixers and nutrient availability (e.g., Berman-Frank et al., 2001; Sañudo-Wilhelmy et al., 2001; Moore et al., 2009; Chappell et al., 2012; Held et al., 2020). These studies focus on the nutrients limiting nitrogen fixation, but rarely simultaneously look at nutrient limitation of non-diazotrophs within the community. Therefore, they can demonstrate the likely importance of CIC in the context of nitrogen fixation but are unable to identify the underlying case of CIC.

As is evident from these examples, the identification of CIC and its associated case is a difficult task requiring assessments of nutritional status across the entire microbial community. However, as we will describe below, the ability to identify the underlying mechanisms behind instances of co-limitation may improve our ability to predict and monitor the impact CIC has on larger biogeochemical cycles in the present and future ocean.

## Discussion

### Community Interaction Co-limitation Influence on Biogeochemical Cycles

A major goal in modern marine biogeochemistry is to develop a predictive understanding of the relationships between microbial communities and biogeochemistry (Boyd et al., 2015; Moore et al., 2018; Dutkiewicz et al., 2020; Van de Waal and Litchman, 2020). Predicting future changes in such processes as primary production and carbon export to the deep ocean is a challenging task due to the large number of controlling factors (e.g., nutrient supply, temperature) and large uncertainties in the future trajectories of these variables. However, it is widely acknowledged that community composition and diversity, particularly size class distributions and the abundance of different functional groups, play a large role in determining primary production, nutrient cycling, and aspects of the biological carbon pump (Finkel et al., 2010; Tréguer et al., 2018). The studies listed in section “Evidence of Community Interaction Co-limitation in the Literature” have documented instances where community productivity and composition are constrained by the biological production of resources like cobalamin. While it can and has been argued that whatever limits the production of that biologically produced resource is the true limiting nutrient (Karl, 2000), without understanding the production, exchange and susceptibility to change of that biological produced nutrient, we risk remaining in the dark about important controls on microbial community productivity and limiting our capacity to predict responses to change. Using cobalamin as an example, we highlight the potential role CIC may play in global biogeochemical processes.

Cobalamin’s impact on phytoplankton size distributions is one example of the biogeochemical consequences of BPNs. Phytoplankton cell size is often controlled by nutrient availability, with large phytoplankton such as diatoms typically dominating in nutrient-rich regions, and smaller phytoplankton species dominating in oligotrophic waters (Finkel et al., 2010). Effects of cell size on biogeochemical cycles are partly a result of differences in sinking velocity and impacts on grazer community (Finkel et al., 2010; Ward et al., 2012). For example, large cells sink faster than small cells and therefore may contribute more to the biological carbon pump (Ward et al., 2012). Several studies have suggested that high cobalamin availability favors the growth of larger phytoplankton (>5 μm) perhaps because diatoms and dinoflagellates are more likely than other groups to be cobalamin auxotrophs and have many larger representatives (Croft et al., 2005; Sañudo-Wilhelmy et al., 2006; Gobler et al., 2007; Bertrand et al., 2015). In contrast, cyanobacteria may gain competitive advantage in low cobalamin environments because they produce and use their own cobalamin-like molecule, pseudo-cobalamin (Bertrand and Allen, 2012; Helliwell et al., 2016; Heal et al., 2017). This suggests that cobalamin availability may drive changes in size classes and functional groups that could influence contribution to the biological carbon pump. Identifying the mechanisms and interactions that underpin how BPNs impact community composition could help further determine CIC’s role in biogeochemical cycles.

Cobalamin CIC also influences global biogeochemical processes by controlling primary production, and, potentially, algal bloom development. A study at the Spanish Coast, which provides high temporal resolution data across all four seasons, cobalamin was shown to co-limit phytoplankton growth in combination with inorganic nutrients in March. We hypothesized earlier that this scenario likely represents Case B, where cobalamin production is limited by DOM. This period of co-limitation was followed by the transition to cobalamin being the primary limiting nutrient for phytoplankton growth in April at the onset of the spring bloom (most productive period of the year is observed in May), when inorganic nutrients are high and bacterial counts low (Barber-Lluch et al., 2019). This could suggest that although limitation by inorganic nutrients in April was partly relieved, phytoplankton remained cobalamin limited. In turn, the limited phytoplankton growth and DOM production continued to restrict cobalamin production, thereby hindering the community’s ability to quickly recover from nutrient limitation. Although an argument could be made that non-auxotrophic phytoplankton growth may eventually relieve DOM limitation of cobalamin producers, it is unclear how the enhanced non-auxotrophic phytoplankton growth, and subsequent DOM production, affect the timing and strength of cobalamin production. This suggests that the strength and mechanisms of the cobalamin feedback loop affected community productivity and succession over the span of weeks. Furthermore, cobalamin limitation in spring may result in a shift in phytoplankton community to the dominance of species with cobalamin-independent growth at the onset of the bloom. Perhaps due to higher resource use costs for cobalamin-independent growth (Bertrand et al., 2013), this may consequently result in a time-lag in maximal productivity and an increased nutrient consumption, hence decreasing the overall productivity of a bloom. Investigating the spatio-temporal patterns of CIC, especially during periods of high productivity, could uncover additional mechanisms of CIC and its influence on global primary production in the ocean, now and in the future.

### Community Interaction Co-limitation in the Future Ocean

Considering the controls of CIC and biological produced nutrients could prove important when predicting the impact of on-going climatic change. Assessing the direct impact on CIC is challenging, due to large uncertainties in predicting the spatial extent and intensity of such environmental changes, and the limited understanding of potential biological responses to them. However, an improved understanding of the relationships between key environmental variables and BPNs might be a first step to anticipate the fate of CIC in the future ocean. Here we explore the possible impact predicted environmental conditions might have on cobalamin availability and requirements, and their potential consequences for CIC.

Cobalamin availability, and instances of cobalamin CIC, might be influenced by changes in light, nutrient supply and sea surface temperature (SST) (Bopp et al., 2001, 2013; Sarmiento et al., 2004a,b; Doney et al., 2012). An increase in irradiance is predicted to occur in many ocean regions due to enhanced stratification and sea-ice retreat (Grebmeier et al., 2010; Doney et al., 2012). This could have a negative impact on the availability of cobalamin by increasing its photodegradation (Juzeniene and Nizauskaite, 2013), thereby enhancing the potential for cobalamin limitation and CIC. One factor that could influence the patterns of CIC in the future ocean is the predicted changes in the supply of other essential nutrients (Tagliabue et al., 2017; Moore et al., 2018). For example, an increased supply of a limiting nutrient in a given region may result in an increase in productivity and a shift to another nutrient becoming limiting or co-limiting. Changes in SST are likely to have an impact on metabolic rates (Talling, 1955; Eppley, 1972) which could influence cobalamin production rates and competition for cobalamin, or other BPNs, within communities. Labile DOM availability has been suggested to be a more important factor for bacterial production than temperature in polar regions (Kirchman et al., 2009) indicating tightly coupled changes in metabolic rates of phytoplankton and bacteria. Other studies have suggested a larger increase in bacterial activity and remineralization rates compared to photosynthesis rates with increasing temperature (Lopez-Urrutia et al., 2006) suggesting a larger increase in cobalamin production compared to consumption by phytoplankton, thereby reducing the potential for cobalamin limitation. However, enhanced bacterial growth could also result in increased competition for cobalamin, as auxotrophic bacterial growth will likely also positively correlate with temperature. Overall, the response of cobalamin CIC will be a result of the balance between the relative response of cobalamin producers, phytoplankton growth and (competing) bacterial cobalamin consumers, as well as how their nutrient production rates and cellular quotas/nutrient demand changes. Few relationships and feedback mechanisms of CIC are identified to date and a more detailed understanding of the effects on community interactions is required. Therefore, predicting the response of CIC to environmental changes might benefit from incorporating ecological and evolutionary theories that aim to understand microbial interactions and dependencies. Furthermore, considering evolutionary forces underpinning microbial interactions may streamline evaluation of possible adaptations of microbial communities to environmental change in the future.

### Incorporating Ecological and Evolutionary Theory Into Community Interaction Co-limitation

The idea that microbial communities are more than the sum of the species present, due to the intricate web of interactions between community members, is foundational to the concept of CIC. Microbial interactions underpin productivity, composition, and resilience in microbial communities, factors that determine a community’s contribution to larger ecological processes. To date, the field studies of nutrient co-limitation that lead us to hypothesize that CIC may play an important role in larger biogeochemical cycles are largely focused on bulk biogeochemical parameters and processes: by and large, the measurements and principles applied in such studies fail to examine the elaborate interactions in marine microbial communities. To attempt to elucidate the experience, and response, of microbial communities under nutrient co-limitation, additional steps must be taken that understand the potential ecological and evolutionary processes at play. Ultimately, this could help to streamline identification of key relationships and underpinning interactions and begin to systematically investigate the factors that influence them.

Microbial interactions are ubiquitous in the marine environment and are usually classified into categories such as mutualism, commensalism, and competition (Ramanan et al., 2016). However, these associations are extremely dynamic and can quickly switch from cooperation to competition, and vice versa, depending on an organism’s metabolic status and nutrient availability (Seyedsayamdost et al., 2011; Hoek et al., 2016). These associations are not (entirely) random, and it has been shown that specific groups of bacteria and algae tend to co-occur in the marine environment (Zelezniak et al., 2015; Milici et al., 2016). Studies have discovered that phytoplankton, or “hosts,” modulate their associated bacterial communities through excreted metabolites (Fu et al., 2020; Shibl et al., 2020). This is beneficial since the close interactions a phytoplankton supports in its phycosphere could determine its access to resources and impact its physiology, productivity, and survival (Amin et al., 2009; Bolch et al., 2011, 2017). Therefore, clarifying the direction, plasticity, and intensity of microbial interactions during periods of BPN limitation are relevant factors that can be used to identify cases and predict consequences of CIC. A more detailed understanding of when and why close mutualistic interaction are successful strategies would be particularly beneficial in the context of resource limitation: it affects the energy put into nutrient acquisition, ultimately impacting an organism’s productivity and fitness. Exchanging nutrients in a direct interaction could allow more consistent access to resources and therefore stability during periods of nutrient limitation. However, there are risks associated with this strategy as it relies on the fitness and presence of a partner and could be more energetically costly. In contrast, an organism that is scavenging nutrients from the surrounding environment may have more restricted access when a resource becomes scarce. The success of this organism relies on efficient acquisition strategies, as there would most likely be competition for the resource from all the consumers within the community. To draw an example from CIC, organisms that are in direct, mutualistic interaction exchanging cobalamin during times of limitation may out-compete scavengers, which would influence composition and species succession in the community. The prevalence of interaction-based nutrient acquisition strategies within a microbial community should be acknowledged when interpreting instances of CIC as it could identify organisms more vulnerable to CIC (e.g., scavengers) and factors that influence formation of cooperative interactions. Incorporating microbial interactions into resource limitation studies could uncover larger ecological or evolutionary patterns and perspectives regarding when and where these strategies are successful.

Community interaction co-limitation can be viewed as the imbalance of resource supply and demand between groups within a community, which is likely to be impacted by community composition. Therefore, enhanced insight into the ecological principles that control microbial community organization could enable better predictions of the patterns and mechanisms controlling CIC, and their susceptibility to change. Recently, computational modeling at a scale unattainable by culture studies alone, has identified important controls on community assembly (Pacheco et al., 2019). Microbial community assembly appears replicable and can be explained by a mix of environmental factors, nutrient availability, microbial interactions, and metabolite profiles of key species (Friedman et al., 2017; Louca et al., 2017; Goldford et al., 2018; Fu et al., 2020). Cross-feeding is a nutrient-centric type of microbial interaction and is becoming recognized as central control of microbial community assembly (Goldford et al., 2018; Pacheco et al., 2019). Cross-feeding is the transfer of metabolites between organisms that improve the fitness of recipient and is classified into specific types based on reciprocity of exchange (D’Souza et al., 2018; Smith et al., 2019). Until recently, it was assumed the cross-feeding had to have a metabolic cost for the producing organisms, however, it has been demonstrated that metabolites which do not impact an organism’s fitness when excreted (e.g., costless) enrich minimal nutrient environments and support interactions between species (Pacheco et al., 2019).

Analogies can be made between specific types of cross-feeding and cases of CIC, as the BPN is produced by one group of organisms and used by another. For example, Case A of CIC can be compared to a simple case of metabolite cross-feeding where one organism releases a metabolite that is used by another organism (Smith et al., 2019). However, unlike CIC, the original classification of metabolite cross-feeding defined the exchanged metabolite as a waste product that cannot be further metabolized by the producing bacteria. Case B is comparable to mutual, or two way, cross-feeding because of the exchange of both labile carbon and cobalamin between organisms. These analogies are most easily transferrable to a bipartite interaction scale, however, CIC adds an important dimension to cross feeding concepts, as it emphasizes that possibility that “public good” metabolites can limit production at a community level. Interdisciplinary research into cross-feeding and resource limitation could identify other metabolites, or their precursors, that control community production and assembly further identifying instances of CIC. Exploring the labile carbon requirements of cobalamin producers or nitrogen fixers is an exciting avenue for further research that could provide additional insight into Case B of CIC. Systematically integrating community assembly models and theories into research surrounding CIC (and vice versa) would be a powerful step forward in enabling predictions of the prevalence of and mechanisms behind CIC.

Ecological interactions between micro-organisms are the result of millions of years of co-evolution and thus ecological theories cannot be discussed without the appreciation of evolution. Evolutionary theories are being developed to describe how the ecological interactions between micro-organisms emerged and control microbial community productivity and composition. Recent studies have sought to better understand the evolution of microbial interactions using evolutionary game theory, examining the fitness response of individuals that are interacting within a community (Frey, 2010; Hummert et al., 2014). These are particularly powerful when coupled with genome-scale metabolic networks which allow growth predictions about interactions and metabolic exchange based on organism’s genomes (Zomorrodi and Segrè, 2017). Such research is developing theoretical principles, based on evolutionary mechanisms, which could more accurately describe resulting future shifts in the form and function of microbial communities. Incorporating such theories into resource limitation research could provide a framework to anticipate the response of CIC and key microbial interactions in the modern and future ocean.

One evolutionary theory that could be particularly relevant in resource-limited environments is The Black Queen Hypothesis, which describes the evolution of obligate relationships in microbial communities (Morris et al., 2012; Mas et al., 2016). It hypothesizes that “mutualistic” obligate interactions can arise because of leaky production of a “common good,” which could be a nutrient, by the “helper,” which prompts loss of function in another organism, the “beneficiary” (Mas et al., 2016). This produces an obligate interaction between the beneficiary and helper, as the beneficiary is no longer able to produce the common good nutrient for the fitness benefit of a reduced genome (Giovannoni et al., 2014; Mas et al., 2016). This theory is also particularly relevant as it considers the cost of production of a particular “public good” which could be relevant for understanding why such cases of CIC can occur. Investigating the cost of the BPN (production vs. acquisition) could help researchers understand which BPNs are likely to be a limiting nutrient in the environment and factors that influence them. The theory may shed some light on the occurrence and evolutionary benefit of microbial dependencies and nutrient exchange during times of resource limitation. Once the types (e.g., obligate or facultative) of key interactions underpinning instances of CIC are identified, the details of nutrient exchange and the associated feedback loop might become clearer, furthering investigation into factors that influence an interaction’s stability and potentially helping to predict responses in an eco-evolutionary framework. Therefore, systematically incorporating developments in microbial evolution and ecology will support better synthesis, interpretation and prediction of key microbial interactions that underpin cases of CIC.

### Data Gaps, Uncertainties, and Future Directions

While the presence of co-limitation in the ocean is known to be widespread (e.g., around 8% of the surface ocean is estimated to experience co-limitation by nitrogen and iron; Browning et al., 2017), we largely lack a detailed understanding of mechanisms behind co-limitation and its broader effect on global biogeochemical cycles. Studies investigating CIC are scarce to date, resulting in limited knowledge about the variety of biologically produced resources that can lead to CIC. Low spatial and temporal resolution in documented cases of CIC restricts our understanding of the spatial extent and seasonal distribution of CIC and its driving factors. However, understanding the mechanisms and susceptibility of CIC to change in future ocean directly relies on researcher’s ability to detect and identify it. This is not straightforward, as it requires the combination of knowledge on different scales including individual organism’s nutritional requirements, community dynamics, and global patterns of nutrient availability and species distribution.

Bottle incubation bioassay experiments offer a valuable insight into the oceanic distribution of nutrient limitations, despite their limitations (e.g., bottle-induced changes in community composition; failure to capture grazing pressure). These studies often do not fully resolve the type of co-limitation which exists and on their own don’t allow researchers to draw conclusions about the community interactions at play. Identifying the biological nutrient producers and consumers in any given environment is a first step in assessing the supply and demand that constrains nutrient availability. However, these identifications will not always reveal the key interactions or community dynamics underpinning the CIC. As highlighted previously, it is important to understand the role bacteria play in nutrient availability and exchange in microbial communities. As such, an important avenue for further research into CIC is the recognition and incorporation of bacterial community response into resource limitation and nutrient addition bioassays.

Auxotrophy is widespread in the marine environment and could have major implications for microbial community interdependencies and nutrient requirements, yet it is still poorly understood (Johnson et al., 2020). To identify additional types of CIC, and monitor the cases of CIC presented, it is critical to understand the occurrence of auxotrophy in species as well as auxophore (metabolites required by auxotrophs) requirements and the factors that influence them. This includes the occurrence of precursor auxotrophy, a budding field of interest (Paerl et al., 2017, 2018a). Additionally, knowing when a community is dominated by auxotrophs might suggest that a community is more vulnerable to cases of CIC. Finally, having an ongoing atlas of common auxophores and instances of auxotrophies in specific primary producers could be of use for studies of CIC.

Sensitive molecular analysis techniques will be required to investigate the interactions and uncover the feedback loops that are critical for defining and monitoring cases of CIC. A successful analysis would uncover (1) which organisms are stressed for or limited by a BPN, (2) which organisms are producing that nutrient, (3) what is limiting the production/transformation of that nutrient and (4) what the nature of interactions between the relevant microbial groups. Although this task seems daunting, it is becoming more feasible with current advances in multi-layered omics analysis.

One approach to uncover the mechanisms of CIC present is by assessing gene or protein expression profiles through meta-transcriptomic or meta-proteomic analyses during nutrient incubation experiments in field studies. Gene expression analyses could allow the identification of organisms that are stressed for or produce a BPN. For example, through meta-transcriptomics, organisms involved in cobalamin cycle and the feedback loops that controlled cobalamin availability were identified in the Southern Ocean (Case B, cobalamin CIC) (Bertrand et al., 2015). Lab cultures can also be a powerful tool for assessing changes in gene and protein expression under varying growth conditions, including pure cultures and co-cultures of interacting microbial species, and can facilitate the identification of relevant target genes and proteins (Helliwell et al., 2018; Wu et al., 2019). Recently, by incorporating various meta-omics techniques, researchers have already made significant advances in the identification of key mechanisms and metabolites involved with microbial interactions that could lead to additional types of CIC (Amin et al., 2015; Bertrand et al., 2015; Durham et al., 2017; Helliwell et al., 2018; Shibl et al., 2020).

Meta-omics studies can also serve as the basis for developing targeted approaches for detecting and monitoring CIC, for instance through identifying critical metabolites and biomarker genes or protein abundances that indicate different nutrient limitation scenarios (Bertrand et al., 2015; Chappell et al., 2015; Heal et al., 2019; Ustick et al., 2021). Such targeted assays require previous knowledge about producers and consumers present and the specific genes of proteins expression patterns sensitive to certain nutrient limitations. However, they can be used more broadly, as the analysis can be faster, and more sensitive and specific than meta-omics approaches. As such, they are a useful tool for assessing global nutrient limitation and CIC patterns when used alone or in combination with bioassay experiments. For example, the use of biomarker peptides for MetE and CBA1 expression (cobalamin stress markers) can be used to indicate regions of cobalamin stress or limitation in the field (Bertrand et al., 2012, 2013). Other biomarker peptide mapping studies have collected valuable data about the nitrogen, iron, and phosphorus status of marine cyanobacterial communities (Saito et al., 2015). These methods could also be used for identification of organisms responsible for biological nutrient production using proteins involved in biological nutrient synthesis (e.g., CobO protein for cobalamin production). However, there is still a big gap in our knowledge about suitable targets to detect nutrient stress for most marine phytoplankton for known limiting or co-limiting nutrients, particularly BPNs involved in CIC.

Furthermore, putting CIC in a global context requires incorporating such evidence found at the organismal and community level with larger oceanographic trends to identify potential spatio-temporal patterns and driving factors that control the underpinning mechanisms of limitation. To map and predict the consequences of CIC in the modern and future ocean, we must apply interdisciplinary methods and approaches capable of elucidating important feedback loops that control nutrient exchange and availability in microbial communities and key factors that influence them. Recent advances in experimental design and molecular approaches provide a hopeful outlook.

## Conclusion

The interactions within a microbial community can control nutrient availability and can profoundly impact metabolic status of community members. Incorporating community interactions into research on resource (co-)limitation enables the identification of community level processes that affect larger biogeochemical cycles. Defining community interaction co-limitation, CIC, as fourth category of nutrient co-limitation allows researchers to systematically combine the tools and findings of microbial ecology and evolution with resource limitation studies to obtain clearer descriptions of mechanisms of nutrient limitation in the environment. It also provides a consistent language and framework that can be used to describe the relevant processes and interactions, hopefully improving cross-disciplinary communication on these subjects. We suggest that the CIC framework will support the systematic examination of the role community interactions have in resource limitation which, in turn, will support more accurate estimates and predictions of the impact these processes have on global biogeochemical cycles.

## Data Availability Statement

The original contributions presented in the study are included in the article/supplementary material, further inquiries can be directed to the corresponding author/s.

## Author Contributions

EB conceived the study. CB and IR performed the literature review. All authors discussed the concepts and wrote the manuscript.

## Conflict of Interest

The authors declare that the research was conducted in the absence of any commercial or financial relationships that could be construed as a potential conflict of interest.

## Publisher’s Note

All claims expressed in this article are solely those of the authors and do not necessarily represent those of their affiliated organizations, or those of the publisher, the editors and the reviewers. Any product that may be evaluated in this article, or claim that may be made by its manufacturer, is not guaranteed or endorsed by the publisher.

## Footnotes

1 Soto, M. A., Desai, D, LaRoche, J, and Bertrand, E. M. (in review). Cobalamin producers and prokaryotic consumers in the Northwest Atlantic. *Environ. Microbiol.*

## References

[B1] Amin S. A. Green D. H. Hart M. C. Küpper F. C. Sunda W. G. Carrano C. J. (2009). Photolysis of iron–siderophore chelates promotes bacterial–algal mutualism. *Proc. Natl. Acad. Sci. U. S. A.* 106 17071–17076. 10.1073/pnas.0905512106 19805106 PMC2761308

[B2] Amin S. A. Hmelo L. R. van Tol H. M. Durham B. P. Carlson L. T. Heal K. R. (2015). Interaction and signalling between a cosmopolitan phytoplankton and associated bacteria. *Nature* 522 98–101. 10.1038/nature14488 26017307

[B3] Amin S. A. Parker M. S. Armbrust E. V. (2012). Interactions between Diatoms and Bacteria. *Microbiol. Mol. Biol. Rev.* 76 667–684. 10.1128/MMBR.00007-12 22933565 PMC3429620

[B4] Banerjee R. Ragsdale S. W. (2003). The Many Faces of Vitamin B 12: catalysis by Cobalamin-Dependent Enzymes. *Annu. Rev. Biochem.* 72 209–247. 10.1146/annurev.biochem.72.121801.161828 14527323

[B5] Barber-Lluch E. Hernández-Ruiz M. Prieto A. Fernández E. Teira E. (2019). Role of vitamin B12 in the microbial plankton response to nutrient enrichment. *Mar. Ecol. Prog. Ser.* 626 29–42. 10.3354/meps13077

[B6] Berman-Frank I. Cullen J. T. Shaked Y. Sherrell R. M. Falkowski P. G. (2001). Iron availability, cellular iron quotas, and nitrogen fixation in Trichodesmium. *Limnol. Oceanogr.* 46 1249–1260. 10.4319/lo.2001.46.6.1249

[B7] Bertrand E. M. Allen A. E. Dupont C. L. Norden-Krichmar T. M. Bai J. Valas R. E. (2012). Influence of cobalamin scarcity on diatom molecular physiology and identification of a cobalamin acquisition protein. *Proc. Natl. Acad. Sci. U. S. A.* 109 E1762–E1771. 10.1073/pnas.1201731109 22652568 PMC3387067

[B8] Bertrand E. M. Allen A. E. (2012). Influence of vitamin B auxotrophy on nitrogen metabolism in eukaryotic phytoplankton. *Front. Microbiol.* 3:375. 10.3389/fmicb.2012.00375 23091473 PMC3476827

[B9] Bertrand E. M. McCrow J. P. Moustafa A. Zheng H. McQuaid J. B. Delmont T. O. (2015). Phytoplankton–bacterial interactions mediate micronutrient colimitation at the coastal Antarctic sea ice edge. *Proc. Natl. Acad. Sci. U. S. A.* 112 9938–9943. 10.1073/pnas.1501615112 26221022 PMC4538660

[B10] Bertrand E. M. Moran D. M. McIlvin M. R. Hoffman J. M. Allen A. E. Saito M. A. (2013). Methionine synthase interreplacement in diatom cultures and communities: implications for the persistence of B12 use by eukaryotic phytoplankton. *Limnol. Oceanogr.* 58 1431–1450. 10.4319/lo.2013.58.4.1431

[B11] Bertrand E. M. Saito M. A. Rose J. M. Riesselman C. R. Lohan M. C. Noble A. E. (2007). Vitamin B12 and iron colimitation of phytoplankton growth in the Ross Sea. *Limnol. Oceanogr.* 52 1079–1093. 10.4319/lo.2007.52.3.1079

[B12] Bolch C. J. S. Bejoy T. A. Green D. H. (2017). Bacterial Associates Modify Growth Dynamics of the Dinoflagellate Gymnodinium catenatum. *Front. Microbiol.* 8:670. 10.3389/fmicb.2017.00670 28469613 PMC5395609

[B13] Bolch C. J. S. Subramanian T. A. Green D. H. (2011). The Toxic Dinoflagellate Gymnodinium Catenatum (dinophyceae) Requires Marine Bacteria for Growth1. *J. Phycol.* 47 1009–1022. 10.1111/j.1529-8817.2011.01043.x 27020182

[B14] Bonnet S. Dekaezemacker J. Turk-Kubo K. A. Moutin T. Hamersley R. M. Grosso O. (2013). Aphotic N2 Fixation in the Eastern Tropical South Pacific Ocean. *PLoS One* 8:e81265. 10.1371/journal.pone.0081265 24349048 PMC3861260

[B15] Bopp L. Monfray P. Aumont O. Dufresne J.-L. Treut H. L. Madec G. (2001). Potential impact of climate change on marine export production. *Glob. Biogeochem. Cycles* 15 81–99. 10.1029/1999GB001256

[B16] Bopp L. Resplandy L. Orr J. C. Doney S. C. Dunne J. P. Gehlen M. (2013). Multiple stressors of ocean ecosystems in the 21st century: projections with CMIP5 models. *Biogeosciences* 10 6225–6245. 10.5194/bg-10-6225-2013

[B17] Boyd P. W. Lennartz S. T. Glover D. M. Doney S. C. (2015). Biological ramifications of climate-change-mediated oceanic multi-stressors. *Nat. Clim. Change* 5 71–79. 10.1038/nclimate2441

[B18] Browning T. J. Achterberg E. P. Engel A. Mawji E. (2021). Manganese co-limitation of phytoplankton growth and major nutrient drawdown in the Southern Ocean. *Nat. Commun.* 12:884. 10.1038/s41467-021-21122-6 33563991 PMC7873070

[B19] Browning T. J. Achterberg E. P. Rapp I. Engel A. Bertrand E. M. Tagliabue A. (2017). Nutrient co-limitation at the boundary of an oceanic gyre. *Nature* 551 242–246. 10.1038/nature24063 29088696

[B20] Chappell P. D. Moffett J. W. Hynes A. M. Webb E. A. (2012). Molecular evidence of iron limitation and availability in the global diazotroph Trichodesmium. *ISME J.* 6 1728–1739. 10.1038/ismej.2012.13 22402399 PMC3498915

[B21] Chappell P. D. Whitney L. P. Wallace J. R. Darer A. I. Jean-Charles S. Jenkins B. D. (2015). Genetic indicators of iron limitation in wild populations of Thalassiosira oceanica from the northeast Pacific Ocean. *ISME J.* 9 592–602. 10.1038/ismej.2014.171 25333460 PMC4331588

[B22] Collier J. L. Baker K. M. Bell S. L. (2009). Diversity of urea-degrading microorganisms in open-ocean and estuarine planktonic communities. *Environ. Microbiol.* 11 3118–3131. 10.1111/j.1462-2920.2009.02016.x 19659552

[B23] Collier J. L. Lovindeer R. Xi Y. Radway J. C. Armstrong R. A. (2012). Differences in Growth and Physiology of Marine Synechococcus (cyanobacteria) on Nitrate Versus Ammonium Are Not Determined Solely by Nitrogen Source Redox State1. *J. Phycol.* 48 106–116. 10.1111/j.1529-8817.2011.01100.x 27009655

[B24] Croft M. T. Lawrence A. D. Raux-Deery E. Warren M. J. Smith A. G. (2005). Algae acquire vitamin B12 through a symbiotic relationship with bacteria. *Nature* 438 90–93. 10.1038/nature04056 16267554

[B25] D’Souza G. Shitut S. Preussger D. Yousif G. Waschina S. Kost C. (2018). Ecology and evolution of metabolic cross-feeding interactions in bacteria. *Nat. Prod. Rep.* 35 455–488. 10.1039/C8NP00009C 29799048

[B26] Danger M. Daufresne T. Lucas F. Pissard S. Lacroix G. (2008). Does Liebig’s law of the minimum scale up from species to communities? *Oikos* 117 1741–1751. 10.1111/j.1600-0706.2008.16793.x

[B27] Doney S. C. Ruckelshaus M. Emmett Duffy J. Barry J. P. Chan F. English C. A. (2012). Climate Change Impacts on Marine Ecosystems. *Annu. Rev. Mar. Sci.* 4 11–37. 10.1146/annurev-marine-041911-111611 22457967

[B28] Dowling D. P. Croft A. K. Drennan C. L. (2012). Radical Use of Rossmann and TIM Barrel Architectures for Controlling Coenzyme B12 Chemistry. *Annu. Rev. Biophys.* 41 403–427. 10.1146/annurev-biophys-050511-102225 22577824

[B29] Durham B. P. Boysen A. K. Carlson L. T. Groussman R. D. Heal K. R. Cain K. R. (2019). Sulfonate-based networks between eukaryotic phytoplankton and heterotrophic bacteria in the surface ocean. *Nat. Microbiol.* 4 1706–1715. 10.1038/s41564-019-0507-5 31332382

[B30] Durham B. P. Dearth S. P. Sharma S. Amin S. A. Smith C. B. Campagna S. R. (2017). Recognition cascade and metabolite transfer in a marine bacteria-phytoplankton model system. *Environ. Microbiol.* 19 3500–3513. 10.1111/1462-2920.13834 28631440

[B31] Dutkiewicz S. Cermeno P. Jahn O. Follows M. J. Hickman A. E. Taniguchi D. A. A. (2020). Dimensions of marine phytoplankton diversity. *Biogeosciences* 17 609–634. 10.5194/bg-17-609-2020

[B32] Eppley R. W. (1972). Temperature and phytoplankton growth in the sea. *Fish. Bull.* 70 1063–1085.

[B33] Finkel Z. V. Beardall J. Flynn K. J. Quigg A. Rees T. A. V. Raven J. A. (2010). Phytoplankton in a changing world: cell size and elemental stoichiometry. *J. Plankton Res.* 32 119–137. 10.1093/plankt/fbp098

[B34] Frey E. (2010). Evolutionary game theory: theoretical concepts and applications to microbial communities. *Phys. Stat. Mech. Appl.* 389 4265–4298. 10.1016/j.physa.2010.02.047

[B35] Friedman J. Higgins L. M. Gore J. (2017). Community structure follows simple assembly rules in microbial microcosms. *Nat. Ecol. Evol.* 1 1–7. 10.1038/s41559-017-0109 28812687

[B36] Fu H. Uchimiya M. Gore J. Moran M. A. (2020). Ecological drivers of bacterial community assembly in synthetic phycospheres. *Proc. Natl. Acad. Sci. U. S. A.* 117 3656–3662. 10.1073/pnas.1917265117 32015111 PMC7035482

[B37] Garcia N. S. Sexton J. Riggins T. Brown J. Lomas M. W. Martiny A. C. (2018). High Variability in Cellular Stoichiometry of Carbon, Nitrogen, and Phosphorus Within Classes of Marine Eukaryotic Phytoplankton Under Sufficient Nutrient Conditions. *Front. Microbiol.* 9:543. 10.3389/fmicb.2018.00543 29636735 PMC5880891

[B38] Giovannoni S. J. Cameron Thrash J. Temperton B. (2014). Implications of streamlining theory for microbial ecology. *ISME J.* 8 1553–1565. 10.1038/ismej.2014.60 24739623 PMC4817614

[B39] Gobler C. Norman C. Panzeca C. Taylor G. Sañudo-Wilhelmy S. (2007). Effect of B-vitamins (B1, B12) and inorganic nutrients on algal bloom dynamics in a coastal ecosystem. *Aquat. Microb. Ecol.* 49 181–194. 10.3354/ame01132

[B40] Goldford J. E. Lu N. Baji D. Sanchez-Gorostiaga A. Segrè D. Mehta P. (2018). Emergent simplicity in microbial community assembly. *Science* 361 469–474. 10.1126/science.aat1168 30072533 PMC6405290

[B41] Grant M. A. Kazamia E. Cicuta P. Smith A. G. (2014). Direct exchange of vitamin B12 is demonstrated by modelling the growth dynamics of algal–bacterial cocultures. *ISME J.* 8 1418–1427. 10.1038/ismej.2014.9 24522262 PMC4069406

[B42] Grebmeier J. M. Moore S. E. Overland J. E. Frey K. E. Gradinger R. (2010). Biological Response to Recent Pacific Arctic Sea Ice Retreats. *Eos Trans. AGU* 91:161. 10.1029/2010EO180001

[B43] Harpole W. S. Ngai J. T. Cleland E. E. Seabloom E. W. Borer E. T. Bracken M. E. S. (2011). Nutrient co-limitation of primary producer communities. *Ecol. Lett.* 14 852–862. 10.1111/j.1461-0248.2011.01651.x 21749598

[B44] Heal K. R. Carlson L. T. Devol A. H. Armbrust E. V. Moffett J. W. Stahl D. A. (2014). Determination of four forms of vitamin B 12 and other B vitamins in seawater by liquid chromatography/tandem mass spectrometry: analysis of forms of B 12 in seawater. *Rapid Commun. Mass Spectrom.* 28 2398–2404. 10.1002/rcm.7040 25303468

[B45] Heal K. R. Kellogg N. A. Carlson L. T. Lionheart R. M. Ingalls A. E. (2019). Metabolic Consequences of Cobalamin Scarcity in the Diatom Thalassiosira pseudonana as Revealed Through Metabolomics. *Protist* 170 328–348. 10.1016/j.protis.2019.05.004 31260945

[B46] Heal K. R. Qin W. Ribalet F. Bertagnolli A. D. Coyote-Maestas W. Hmelo L. R. (2017). Two distinct pools of B 12 analogs reveal community interdependencies in the ocean. *Proc. Natl. Acad. Sci. U. S. A.* 114 364–369. 10.1073/pnas.1608462114 28028206 PMC5240700

[B47] Held N. A. Webb E. A. McIlvin M. M. Hutchins D. A. Cohen N. R. Moran D. M. (2020). Co-occurrence of Fe and P stress in natural populations of the marine diazotroph <i>Trichodesmium&it;/i&gt. *Biogeosciences* 17 2537–2551. 10.5194/bg-17-2537-2020

[B48] Helliwell K. E. (2017). The roles of B vitamins in phytoplankton nutrition: new perspectives and prospects. *New Phytol.* 216 62–68. 10.1111/nph.14669 28656633

[B49] Helliwell K. E. Lawrence A. D. Holzer A. Kudahl U. J. Sasso S. Kräutler B. (2016). Cyanobacteria and Eukaryotic Algae Use Different Chemical Variants of Vitamin B12. *Curr. Biol.* 26 999–1008. 10.1016/j.cub.2016.02.041 27040778 PMC4850488

[B50] Helliwell K. E. Pandhal J. Cooper M. B. Longworth J. Kudahl U. J. Russo D. A. (2018). Quantitative proteomics of a B12-dependent alga grown in coculture with bacteria reveals metabolic tradeoffs required for mutualism. *New Phytol.* 217 599–612. 10.1111/nph.14832 29034959 PMC5765456

[B51] Helliwell K. E. Wheeler G. L. Leptos K. C. Goldstein R. E. Smith A. G. (2011). Insights into the Evolution of Vitamin B12 Auxotrophy from Sequenced Algal Genomes. *Mol. Biol. Evol.* 28 2921–2933. 10.1093/molbev/msr124 21551270

[B52] Ho T.-Y. Quigg A. Finkel Z. V. Milligan A. J. Wyman K. Falkowski P. G. (2003). The Elemental Composition of Some Marine Phytoplankton1. *J. Phycol.* 39 1145–1159. 10.1111/j.0022-3646.2003.03-090.x

[B53] Hodgkin D. C. Kamper J. Mackay M. Pickworth J. Trueblood K. N. White J. G. (1956). Structure of vitamin B12. *Nature* 178 64–66. 10.1038/178064a0 13348621

[B54] Hoek T. A. Axelrod K. Biancalani T. Yurtsev E. A. Liu J. Gore J. (2016). Resource Availability Modulates the Cooperative and Competitive Nature of a Microbial Cross-Feeding Mutualism. *PLoS Biol.* 14:e1002540. 10.1371/journal.pbio.1002540 27557335 PMC4996419

[B55] Hummert S. Bohl K. Basanta D. Deutsch A. Werner S. Theißen G. (2014). Evolutionary game theory: cells as players. *Mol. BioSyst.* 10 3044–3065. 10.1039/C3MB70602H 25270362

[B56] Hutchins D. A. Witter A. E. Butler A. Luther G. W. (1999). Competition among marine phytoplankton for different chelated iron species. *Nature* 400 858–861. 10.1038/23680

[B57] Johnson W. M. Alexander H. Bier R. L. Miller D. R. Muscarella M. E. Pitz K. J. (2020). Auxotrophic interactions: a stabilizing attribute of aquatic microbial communities? *FEMS Microbiol. Ecol.* 96:fiaa115. 10.1093/femsec/fiaa115 32520336 PMC7609354

[B58] Juzeniene A. Nizauskaite Z. (2013). Photodegradation of cobalamins in aqueous solutions and in human blood. *J. Photochem. Photobiol. B Biol.* 122 7–14. 10.1016/j.jphotobiol.2013.03.001 23558034

[B59] Karl D. M. (2000). Phosphorus, the staff of life. *Nature* 406 31–33. 10.1038/35017683 10894527

[B60] Karl D. Michaels A. Bergman B. Capone D. Carpenter E. Letelier R. (2002). “Dinitrogen fixation in the world’s oceans,” in *The Nitrogen Cycle at Regional to Global Scales*, eds Boyer E. W. Howarth R. W. (Dordrecht: Springer Netherlands), 47–98. 10.1007/978-94-017-3405-9_2

[B61] Kazamia E. Czesnick H. Nguyen T. T. V. Croft M. T. Sherwood E. Sasso S. (2012). Mutualistic interactions between vitamin B12-dependent algae and heterotrophic bacteria exhibit regulation. *Environ. Microbiol.* 14 1466–1476. 10.1111/j.1462-2920.2012.02733.x 22463064

[B62] Kirchman D. L. Morán X. A. G. Ducklow H. (2009). Microbial growth in the polar oceans — role of temperature and potential impact of climate change. *Nat. Rev. Microbiol.* 7 451–459. 10.1038/nrmicro2115 19421189

[B63] Koch F. Hattenrath-Lehmann T. K. Goleski J. A. Sanudo-Wilhelmy S. Fisher N. S. Gobler C. J. (2012). Vitamin B1 and B12 Uptake and Cycling by Plankton Communities in Coastal Ecosystems. *Front. Microbiol.* 3:363. 10.3389/fmicb.2012.00363 23091470 PMC3469840

[B64] Koch F. Marcoval M. A. Panzeca C. Bruland K. W. Sañudo-Wilhelmy S. A. Gobler C. J. (2011). The effect of vitamin B12 on phytoplankton growth and community structure in the Gulf of Alaska. *Limnol. Oceanogr.* 56 1023–1034. 10.4319/lo.2011.56.3.1023

[B65] Landolfi A. Koeve W. Dietze H. Kähler P. Oschlies A. (2015). A new perspective on environmental controls of marine nitrogen fixation. *Geophys. Res. Lett.* 42 4482–4489. 10.1002/2015GL063756

[B66] Lopez-Urrutia A. San Martin E. Harris R. P. Irigoien X. (2006). Scaling the metabolic balance of the oceans. *Proc. Natl. Acad. Sci. U. S. A.* 103 8739–8744. 10.1073/pnas.0601137103 16731624 PMC1482648

[B67] Louca S. Jacques S. M. S. Pires A. P. F. Leal J. S. Srivastava D. S. Parfrey L. W. (2017). High taxonomic variability despite stable functional structure across microbial communities. *Nat. Ecol. Evol.* 1:0015. 10.1038/s41559-016-0015 28812567

[B68] Luo H. Benner R. Long R. A. Hu J. (2009). Subcellular localization of marine bacterial alkaline phosphatases. *Proc. Natl. Acad. Sci. U. S. A.* 106 21219–21223. 10.1073/pnas.0907586106 19926862 PMC2795515

[B69] Ma A. T. Tyrell B. Beld J. (2020). Specificity of cobamide remodeling, uptake and utilization in *Vibrio cholerae*. *Mol. Microbiol.* 113 89–102. 10.1111/mmi.14402 31609521

[B70] Manck L. E. Park J. Tully B. J. Poire A. M. Bundy R. M. Dupont C. L. (2022). Petrobactin, a siderophore produced by Alteromonas, mediates community iron acquisition in the global ocean. *ISME J.* 16 358–369. 10.1038/s41396-021-01065-y 34341506 PMC8776838

[B71] Martens J.-H. Barg H. Warren M. Jahn D. (2002). Microbial production of vitamin B12. *Appl. Microbiol. Biotechnol.* 58 275–285. 10.1007/s00253-001-0902-7 11935176

[B72] Mas A. Jamshidi S. Lagadeuc Y. Eveillard D. Vandenkoornhuyse P. (2016). Beyond the Black Queen Hypothesis. *ISME J.* 10 2085–2091. 10.1038/ismej.2016.22 26953598 PMC4989313

[B73] Milici M. Deng Z.-L. Tomasch J. Decelle J. Wos-Oxley M. L. Wang H. (2016). Co-occurrence Analysis of Microbial Taxa in the Atlantic Ocean Reveals High Connectivity in the Free-Living Bacterioplankton. *Front. Microbiol.* 7:649. 10.3389/fmicb.2016.00649 27199970 PMC4858663

[B74] Mills M. M. Ridame C. Davey M. La Roche J. Geider R. J. (2004). Iron and phosphorus co-limit nitrogen fixation in the eastern tropical North Atlantic. *Nature* 429 292–294. 10.1038/nature02550 15152251

[B75] Moisander P. H. Zhang R. Boyle E. A. Hewson I. Montoya J. P. Zehr J. P. (2012). Analogous nutrient limitations in unicellular diazotrophs and Prochlorococcus in the South Pacific Ocean. *ISME J.* 6 733–744. 10.1038/ismej.2011.152 22094348 PMC3309360

[B76] Moore C. M. Mills M. M. Achterberg E. P. Geider R. J. LaRoche J. Lucas M. I. (2009). Large-scale distribution of Atlantic nitrogen fixation controlled by iron availability. *Nat. Geosci.* 2 867–871. 10.1038/ngeo667

[B77] Moore C. M. Mills M. M. Arrigo K. R. Berman-Frank I. Bopp L. Boyd P. W. (2013). Processes and patterns of oceanic nutrient limitation. *Nat. Geosci.* 6 701–710. 10.1038/ngeo1765

[B78] Moore J. K. Fu W. Primeau F. Britten G. L. Lindsay K. Long M. (2018). Sustained climate warming drives declining marine biological productivity. *Science* 359 1139–1143. 10.1126/science.aao6379 29590043

[B79] Moore L. R. Post A. F. Rocap G. Chisholm S. W. (2002). Utilization of different nitrogen sources by the marine cyanobacteria Prochlorococcus and Synechococcus. *Limnol. Oceanogr.* 47 989–996. 10.4319/lo.2002.47.4.0989

[B80] Morris J. J. Lenski R. E. Zinser E. R. (2012). The Black Queen Hypothesis: evolution of Dependencies through Adaptive Gene Loss. *mBio* 3 e00036–12. 10.1128/mBio.00036-12 22448042 PMC3315703

[B81] Muñoz-Marín M. del C. Shilova I. N. Shi T. Farnelid H. Cabello A. M. (2018). The Transcriptional Cycle Is Suited to Daytime N 2 Fixation in the Unicellular Cyanobacterium “Candidatus Atelocyanobacterium thalassa” (UCYN-A). *mBio* 10 e02495–18. 10.1128/mBio.02495-18 30602582 PMC6315102

[B82] Noble A. E. Lamborg C. H. Ohnemus D. C. Lam P. J. Goepfert T. J. Measures C. I. (2012). Basin-scale inputs of cobalt, iron, and manganese from the Benguela-Angola front to the South Atlantic Ocean. *Limnol. Oceanogr.* 57 989–1010. 10.4319/lo.2012.57.4.0989

[B83] Pacheco A. R. Moel M. Segrè D. (2019). Costless metabolic secretions as drivers of interspecies interactions in microbial ecosystems. *Nat. Commun.* 10:103. 10.1038/s41467-018-07946-9 30626871 PMC6327061

[B84] Paerl R. W. Bertrand E. M. Rowland E. Schatt P. Mehiri M. Niehaus T. D. (2018a). Carboxythiazole is a key microbial nutrient currency and critical component of thiamin biosynthesis. *Sci. Rep.* 8:5940. 10.1038/s41598-018-24321-2 29654239 PMC5899164

[B85] Paerl R. W. Sundh J. Tan D. Svenningsen S. L. Hylander S. Pinhassi J. (2018b). Prevalent reliance of bacterioplankton on exogenous vitamin B1 and precursor availability. *Proc. Natl. Acad. Sci. U. S. A.* 115 E10447–E10456. 10.1073/pnas.1806425115 30322929 PMC6217396

[B86] Paerl R. W. Bouget F.-Y. Lozano J.-C. Vergé V. Schatt P. Allen E. E. (2017). Use of plankton-derived vitamin B1 precursors, especially thiazole-related precursor, by key marine picoeukaryotic phytoplankton. *ISME J.* 11 753–765. 10.1038/ismej.2016.145 27935586 PMC5322297

[B87] Panzeca C. Beck A. J. Leblanc K. Taylor G. T. Hutchins D. A. Sañudo-Wilhelmy S. A. (2008). Potential cobalt limitation of vitamin B12 synthesis in the North Atlantic Ocean. *Glob. Biogeochem. Cycles* 22:GB2029. 10.1029/2007GB003124

[B88] Panzeca C. Tovar-Sanchez A. Agustí S. Reche I. Duarte C. M. Taylor G. T. (2006). B vitamins as regulators of phytoplankton dynamics. *Eos Trans. Am. Geophys. Union* 87 593–596. 10.1029/2006EO520001

[B89] Rahav E. Giannetto M. J. Bar-Zeev E. (2016). Contribution of mono and polysaccharides to heterotrophic N2 fixation at the eastern Mediterranean coastline. *Sci. Rep.* 6:27858. 10.1038/srep27858 27306501 PMC4910064

[B90] Rahav E. Herut B. Mulholland M. Belkin N. Elifantz H. Berman-Frank I. (2015). Heterotrophic and autotrophic contribution to dinitrogen fixation in the Gulf of Aqaba. *Mar. Ecol. Prog. Ser.* 522 67–77. 10.3354/meps11143

[B91] Ramanan R. Kim B.-H. Cho D.-H. Oh H.-M. Kim H.-S. (2016). Algae–bacteria interactions: evolution, ecology and emerging applications. *Biotechnol. Adv.* 34 14–29. 10.1016/j.biotechadv.2015.12.003 26657897

[B92] Saito M. A. Dorsk A. Post A. F. McIlvin M. R. Rappé M. S. DiTullio G. R. (2015). Needles in the blue sea: sub-species specificity in targeted protein biomarker analyses within the vast oceanic microbial metaproteome. *Proteomics* 15 3521–3531. 10.1002/pmic.201400630 26097212

[B93] Saito M. A. Goepfert T. J. Ritt J. T. (2008). Some thoughts on the concept of colimitation: three definitions and the importance of bioavailability. *Limnol. Oceanogr.* 53 276–290. 10.4319/lo.2008.53.1.0276

[B94] Sanchez N. Brown E. A. Olsen Y. Vadstein O. Iriarte J. L. Gonzalez H. E. (2018). Effect of Siderophore on Iron Availability in a Diatom and a Dinoflagellate Species: contrasting Response in Associated Bacteria. *Front. Mar. Sci.* 5:118. 10.3389/fmars.2018.00118

[B95] Sañudo-Wilhelmy S. A. Gobler C. J. Okbamichael M. Taylor G. T. (2006). Regulation of phytoplankton dynamics by vitamin B12. *Geophys. Res. Lett.* 33 1–4. 10.1029/2005GL025046

[B96] Sañudo-Wilhelmy S. A. Gómez-Consarnau L. Suffridge C. Webb E. A. (2014). The Role of B Vitamins in Marine Biogeochemistry. *Annu. Rev. Mar. Sci.* 6 339–367. 10.1146/annurev-marine-120710-100912 24050603

[B97] Sañudo-Wilhelmy S. A. Kustka A. B. Gobler C. J. Hutchins D. A. Yang M. Lwiza K. (2001). Phosphorus limitation of nitrogen fixation by Trichodesmium in the central Atlantic Ocean. *Nature* 411 66–69. 10.1038/35075041 11333977

[B98] Sarmiento J. L. Gruber N. Brzezinski M. A. Dunne J. P. (2004a). High-latitude controls of thermocline nutrients and low latitude biological productivity. *Nature* 427 56–60. 10.1038/nature02127 14702082

[B99] Sarmiento J. L. Slater R. Barber R. Bopp L. Doney S. C. Hirst A. C. (2004b). Response of ocean ecosystems to climate warming. *Glob. Biogeochem. Cycles* 18:GB3003. 10.1029/2003GB002134

[B100] Seyedsayamdost M. R. Case R. J. Kolter R. Clardy J. (2011). The Jekyll-and-Hyde chemistry of Phaeobacter gallaeciensis. *Nat. Chem.* 3 331–335. 10.1038/nchem.1002 21430694 PMC3376411

[B101] Seymour J. R. Amin S. A. Raina J.-B. Stocker R. (2017). Zooming in on the phycosphere: the ecological interface for phytoplankton-bacteria relationships. *Nat. Microbiol.* 2, 17065. 10.1038/nmicrobiol.2017.65 28555622

[B102] Shelton A. N. Seth E. C. Mok K. C. Han A. W. Jackson S. N. Haft D. R. (2019). Uneven distribution of cobamide biosynthesis and dependence in bacteria predicted by comparative genomics. *ISME J.* 13 789–804. 10.1038/s41396-018-0304-9 30429574 PMC6461909

[B103] Shibl A. A. Isaac A. Ochsenkühn M. A. Cárdenas A. Fei C. Behringer G. (2020). Diatom modulation of select bacteria through use of two unique secondary metabolites. *Proc. Natl. Acad. Sci. U. S. A.* 117 27445–27455. 10.1073/pnas.2012088117 33067398 PMC7959551

[B104] Soto M. A. Desai D LaRoche J Bertrand E. M. (in review). Cobalamin producers and prokaryotic consumers in the Northwest Atlantic. *Environ. Microbiol.* 10.1111/1462-2920.16363 36861357

[B105] Smith N. W. Shorten P. R. Altermann E. Roy N. C. McNabb W. C. (2019). The Classification and Evolution of Bacterial Cross-Feeding. *Front. Ecol. Evol.* 7:153. 10.3389/fevo.2019.00153

[B106] Sohm J. A. Webb E. A. Capone D. G. (2011). Emerging patterns of marine nitrogen fixation. *Nat. Rev. Microbiol.* 9 499–508. 10.1038/nrmicro2594 21677685

[B107] Sokolovskaya O. M. Shelton A. N. Taga M. E. (2020). Sharing vitamins: cobamides unveil microbial interactions. *Science* 369:eaba0165. 10.1126/science.aba0165 32631870 PMC8654454

[B108] Sutak R. Camadro J.-M. Lesuisse E. (2020). Iron Uptake Mechanisms in Marine Phytoplankton. *Front. Microbiol.* 11:566691. 10.3389/fmicb.2020.566691 33250865 PMC7676907

[B109] Tagliabue A. Bowie A. R. Boyd P. W. Buck K. N. Johnson K. S. Saito M. A. (2017). The integral role of iron in ocean biogeochemistry. *Nature* 543 51–59. 10.1038/nature21058 28252066

[B110] Tagliabue A. Hawco N. J. Bundy R. M. Landing W. M. Milne A. Morton P. L. (2018). The Role of External Inputs and Internal Cycling in Shaping the Global Ocean Cobalt Distribution: insights From the First Cobalt Biogeochemical Model. *Glob. Biogeochem. Cycles* 32 594–616. 10.1002/2017GB005830 29937626 PMC5993222

[B111] Talling J. F. (1955). The Relative Growth Rates of Three Plankton Diatoms in Relation to Underwater Radiation and Temperature. *Ann. Bot.* 19 329–341. 10.1093/oxfordjournals.aob.a083432

[B112] Thompson A. W. Foster R. A. Krupke A. Carter B. J. Musat N. Vaulot D. (2012). Unicellular Cyanobacterium Symbiotic with a Single-Celled Eukaryotic Alga. *Science* 337 1546–1550. 10.1126/science.1222700 22997339

[B113] Tréguer P. Bowler C. Moriceau B. Dutkiewicz S. Gehlen M. Aumont O. (2018). Influence of diatom diversity on the ocean biological carbon pump. *Nat. Geosci.* 11 27–37. 10.1038/s41561-017-0028-x

[B114] Twining B. S. Baines S. B. (2013). The Trace Metal Composition of Marine Phytoplankton. *Annu. Rev. Mar. Sci.* 5 191–215. 10.1146/annurev-marine-121211-172322 22809181

[B115] Twining B. S. Antipova O. Chappell P. D. Cohen N. R. Jacquot J. E. Mann E. L. (2021). Taxonomic and nutrient controls on phytoplankton iron quotas in the ocean. *Limnol. Oceanogr. Lett.* 6 96–106. 10.1002/lol2.10179

[B116] Ustick L. J. Larkin A. A. Garcia C. A. Garcia N. S. Brock M. L. Lee J. A. (2021). Metagenomic analysis reveals global-scale patterns of ocean nutrient limitation. *Science* 372 287–291. 10.1126/science.abe6301 33859034

[B117] Van de Waal D. B. Litchman E. (2020). Multiple global change stressor effects on phytoplankton nutrient acquisition in a future ocean. *Philos. Trans. R. Soc. B* 375:20190706. 10.1098/rstb.2019.0706 32200734 PMC7133525

[B118] Ward B. A. Dutkiewicz S. Jahn O. Follows M. J. (2012). A size-structured food-web model for the global ocean. *Limnol. Oceanogr.* 57 1877–1891. 10.4319/lo.2012.57.6.1877

[B119] Wu M. McCain J. S. P. Rowland E. Middag R. Sandgren M. Allen A. E. (2019). Manganese and iron deficiency in Southern Ocean Phaeocystis antarctica populations revealed through taxon-specific protein indicators. *Nat. Commun.* 10:3582. 10.1038/s41467-019-11426-z 31395884 PMC6687791

[B120] Zehr J. P. Capone D. G. (2020). Changing perspectives in marine nitrogen fixation. *Science* 368:eaay9514. 10.1126/science.aay9514 32409447

[B121] Zelezniak A. Andrejev S. Ponomarova O. Mende D. R. Bork P. Patil K. R. (2015). Metabolic dependencies drive species co-occurrence in diverse microbial communities. *Proc. Natl. Acad. Sci. U. S. A.* 112 6449–6454. 10.1073/pnas.1421834112 25941371 PMC4443341

[B122] Zomorrodi A. R. Segrè D. (2017). Genome-driven evolutionary game theory helps understand the rise of metabolic interdependencies in microbial communities. *Nat. Commun.* 8:1563. 10.1038/s41467-017-01407-5 29146901 PMC5691134

